# Age-Related Patterns in Pediatric Road Traffic Injuries in Romania

**DOI:** 10.3390/jcm14186633

**Published:** 2025-09-20

**Authors:** Ștefan Popa, Carmen Iulia Ciongradi, Adrian Onisim Surd, Ioan Sârbu, Iuliana-Laura Candussi, Irene Paula Popa

**Affiliations:** 1 2nd Department of Surgery—Pediatric Surgery and Orthopedics, “Grigore T. Popa” University of Medicine and Pharmacy Iași, 700115 Iași, Romania; stefan.popa@umfiasi.ro (Ș.P.); carmen.ciongradi@umfiasi.ro (C.I.C.); 2 Department of Pediatric Surgery, “Iuliu Hațieganu” University of Medicine and Pharmacy, 400347 Cluj-Napoca, Romania; adisurd@elearn.umfcluj.ro; 3 Clinical Surgery Department, Faculty of Medicine and Pharmacy, “Dunărea de Jos” University, 800008 Galați, Romania; iuliana.candussi@ugal.ro; 4 Department of Physiology, “Grigore T. Popa” University of Medicine and Pharmacy, 700115 Iași, Romania; irene-paula_popa@umfiasi.ro

**Keywords:** road traffic accidents, children, adolescents, associated trauma

## Abstract

**Background:** Pediatric road traffic injuries (RTIs) represent a significant public health concern, particularly in countries like Romania, where road infrastructure and safety remain challenges. Despite recent economic reclassification, Romania continues to report high rates of pediatric traffic-related injuries. Non-fatal RTIs often result in long-term physical and psychological harm. This study aims to assess age- and gender-specific injury patterns and mechanisms of non-fatal RTIs in children and adolescents, using data from “St. Mary’s” Emergency Clinical Hospital for Children in Iași over a ten-year period to inform targeted prevention strategies. **Methods:** This 10-year retrospective study (2015–2024) was conducted at “St. Mary’s” Emergency Clinical Hospital for Children in Iași, Romania, a regional referral center. Data from 1074 pediatric patients (aged 1 month–17 years, 11 months) with RTIs were analyzed using ICD-10 codes and verified manually. Variables included demographics, injury type, mechanism, and treatment. Patients were stratified into four age groups. Statistical analysis was performed using IBM SPSS Statistics 25, with significance set at *p* < 0.05. **Results:** The highest incidence was observed among boys (77.7%) and children aged 10–14 years. Car passengers and cyclists constituted the most frequently affected groups, with only 11% of passengers appropriately restrained and 78% of cyclists not wearing helmets. Common injuries included excoriations, thoracic contusions, and abdominal trauma, with notable variations by age and sex. Thoracic injuries were more frequent among girls, whereas younger children exhibited a higher incidence of abdominal trauma. **Conclusions:** The findings emphasize critical safety gaps in child restraint and helmet use and highlight the urgent need for targeted, age-specific road safety interventions and improved public health education.

## 1. Introduction

Pediatric road traffic injuries (RTIs) remain a pressing global concern, with profound implications for child health, public safety, and socioeconomic development. According to the United Nations International Children’s Emergency Fund (UNICEF), in 2022 nearly 181,453 individuals under the age of 20 lost their lives due to road-traffic incidents, with over 90% of these fatalities occurring in low- and middle-income countries [1]. Romania, initially classified as an upper-middle-income country in 2020 [2], was later reclassified by the World Bank as a high-income nation in 2021 and 2022 [3], yet it continues to face persistent challenges in addressing pediatric traffic trauma.

The World Health Organization (WHO) estimates that 20 to 50 million people globally suffer non-fatal injuries annually as a result of road collisions, with many cases resulting in long-term or permanent impairments [4]. These figures underscore the reality that fatalities represent only a fraction of the total burden, as non-fatal injuries often impose lasting physical, psychological, and socioeconomic consequences. In fact, the economic cost of traffic-related incidents is staggering—estimated at USD 518 billion annually—representing up to 3% of GDP in certain regions due to healthcare costs, loss of productivity, and prolonged disability care [5].

RTIs are increasingly viewed as emblematic of the so-called “diseases of development,” driven by surging vehicle numbers, population growth, and rapidly transforming urban environments [6]. By 2025, traffic-related injuries are expected to become the third-leading contributor to global disease burden, while their total impact is projected to rank fourth overall [7].

One of the primary contributors to high injury rates is inadequate road infrastructure. Romania consistently receives poor evaluations regarding road quality. In 2022, for example, the World Economic Forum rated its infrastructure at just 3.0 out of 7—among the lowest in Europe—highlighting an urgent need for investment and reform [8]. This infrastructural deficit partially explains the country’s elevated rates of traffic injuries and fatalities among children.

Despite often being labeled as minor, non-fatal injuries can result in severe physical and psychological outcomes that diminish long-term well-being and impose substantial burdens on families and communities [9,10]. Children, in particular, are highly vulnerable in traffic environments. Although they may possess sufficient motor coordination to move through public spaces, they frequently lack the cognitive, perceptual, and behavioral maturity necessary to accurately assess traffic risks [11]. Pedestrian injuries, which are prevalent among children in many countries, frequently lead to more severe trauma than incidents involving child passengers inside vehicles [12,13,14].

The trauma profiles of pediatric patients also differ from those of adults due to anatomical and physiological factors. Lower body mass results in reduced kinetic energy upon impact, and their typical seating position in the rear of vehicles offers added protection. As a result, children generally experience fewer thoracic, abdominal, pelvic, and long bone injuries and often present with lower Injury Severity Scores (ISSs), despite potentially high Glasgow Coma Scale scores [15,16]. Moreover, children’s greater biological resilience enhances their capacity to recover without long-term sequelae; however, when permanent outcomes do occur, medico-legal assessment—such as Personal Injury Assessment (PIA)—is complicated by the unpredictability of future developmental needs [15].

The purpose of this study is to assess age- and gender-specific patterns of associated injuries caused by different mechanisms of non-fatal RTIs in children and adolescents, based on cases presented at the Emergency Department and the Pediatric Orthopedics Department of “St. Mary’s” Emergency Clinical Hospital for Children in Iași, Romania. In alignment with the existing literature, the pediatric population was categorized into four age groups to facilitate comparative analysis of injury trends across developmental stages [15,17].

## 2. Materials and Methods

This retrospective, observational study was conducted over a 10-year period, from 2015 to 2024, at the Emergency Department (ED) and the Pediatric Orthopedics Department of “St. Mary’s” Emergency Clinical Hospital for Children in Iași, Romania.

The hospital serves as a regional referral center for pediatric trauma, providing care to both urban and rural populations across Iași County and the seven surrounding counties of northeastern Romania. The ED receives approximately 40,000 visits annually, with around 4% of these involving trauma cases. Around 54% of those trauma cases affect children between the ages of 7 and 14 years. Between 98 and 127 pediatric RTIs are recorded at the hospital each year, with an average of 102 cases annually.

Patients eligible for inclusion in the study were aged between 1 month and 17 years and 11 months and had sustained injuries resulting from road traffic injuries, with documented presentation to the Emergency Department and subsequent hospitalization in the Pediatric Orthopedics Department. Relevant data were extracted from the hospital’s electronic medical record system, primarily based on ICD-10 external cause codes V01–V99, among which the most frequently identified were V03.1, V06.1, V01.1, V10.3, V10.4, V10.5, V10.9, V13.3, V13.4, V13.5, V13.9, V19.4, V19.5, V19.9, V40.09, V43.03, V47.03, V80.00, V80.09, V80.1, and V99.

To minimize case omission, additional searches were performed using the keywords “road” and “traffic injury.” A manual review was conducted to confirm each case’s relevance, based on the study’s definition of an RTI as a road incident involving at least one moving vehicle. Injuries not related to moving vehicles or to road traffic events were excluded.

A total of 1074 pediatric patients met the inclusion criteria and were included in the analysis. Data collected included demographic information (age and sex), the mechanism and context of the injury, the anatomical region and type of injury, laboratory and imaging investigations, and the treatment approach (conservative or surgical). Injuries were classified based on the child’s role or location at the time of the injury (e.g., pedestrian, cyclist, passenger, and other types of injuries that include a small number of patients who sustained falls from a horse by traffic injury or were in a wagon pulled by horses implicated in a traffic injury), while injuries were classified by anatomical location and nature.

Injury categories were standardized for consistency and included excoriations, defined as superficial skin abrasions not involving deeper tissue; thoracic contusions, referring to chest wall soft tissue injuries (bruising of skin, subcutaneous tissue, and musculature) without confirmed internal organ involvement such as pulmonary or cardiac contusions; abdominal contusions, encompassing both abdominal wall soft tissue injuries and, when applicable, internal organ contusions involving the liver, spleen, or other abdominal viscera; additional fractures, representing bone fractures beyond the primary injury, either multiple fractures within the same limb or simultaneous fractures in different anatomical regions; traumatic brain injuries (TBIs), including documented concussions, intracranial hemorrhage, or skull fractures confirmed by clinical or imaging evaluation; limb contusions, characterized as localized bruising of the extremities without associated fracture; and hematologic alterations, defined as blood count abnormalities, primarily decreases in hemoglobin and hematocrit indicative of blood loss.

Patients were stratified into four age categories: 0–4 years (early childhood), 5–9 years (middle childhood), 10–14 years (early adolescence), and 15–17 years (late adolescence).

Statistical analyses were performed using IBM SPSS Statistics (Version 25) (IBM Corp., Armonk, NY, USA). Descriptive statistics for continuous variables included mean, median, standard deviation, and range (minimum and maximum values). For continuous variables, comparisons were made using Mann–Whitney U test and the Kruskal–Wallis H test. Categorical variables were reported as absolute and relative frequencies, with comparisons made using Fisher’s exact test. A *p*-value < 0.05 was considered statistically significant.

The study was conducted in accordance with the principles of the Declaration of Helsinki and was approved by the Ethics Committees of both “St. Mary’s” Emergency Clinical Hospital for Children and the “Grigore T. Popa” University of Medicine and Pharmacy in Iași. Informed consent was obtained from all legal guardians at the time of admission. Consent forms authorized the use of medical data for research and educational purposes, approved the collection of biological samples and application of necessary diagnostic and therapeutic procedures, and granted permission for photo or video documentation for scientific use, with measures taken to ensure anonymity. All data were fully anonymized, excluding any personal identifiers such as national identification numbers, photos, or diagnostic reports that could lead to patient identification. Data handling complied with EU Regulation 2016/679 (GDPR) to protect patient privacy and confidentiality.

## 3. Results

### 3.1. Demographic Characteristics

The average age among all cases was 11.1 ± 4.4 years, with a median of 12 years. The largest proportion of cases fell within the 10–14 year age group (45.5%), followed by those aged 15–17 (23.7%). A clear majority of the patients were male, representing 77.7% of the total. Statistical analysis showed no significant differences between males and females in terms of age, whether treated as a continuous or categorical variable (*p* > 0.05). Most patients were between 10 and 14 years of age, with 52.1% of females and 45.2% of males falling into this range. For both sexes, the median age remained consistent at 12 years.

Among the cases analyzed, 15 children were under the age of 1 at the time of data collection, comprising 14 boys and 1 girl. Based on the box plot analysis, the distribution of age in the male group extended toward the lower age range (median = 12 years, IQR = 8–14 years). Given that the lower threshold for detecting outliers resulted in no boys under 1 year being flagged as outliers, this age category was not considered statistically significant. In contrast, the single female patient under 1 year of age was classified as an outlier.

### 3.2. Urban–Rural Distribution

There was a statistically significant difference in the distribution of sex across residential settings (*p* = 0.005), with a higher proportion of male patients in rural areas compared to females (79.5% vs. 63.7%), and a higher proportion of females in urban areas compared to males (36.3% vs. 20.5%).

### 3.3. Accident Type by Age

**Figure 1:**
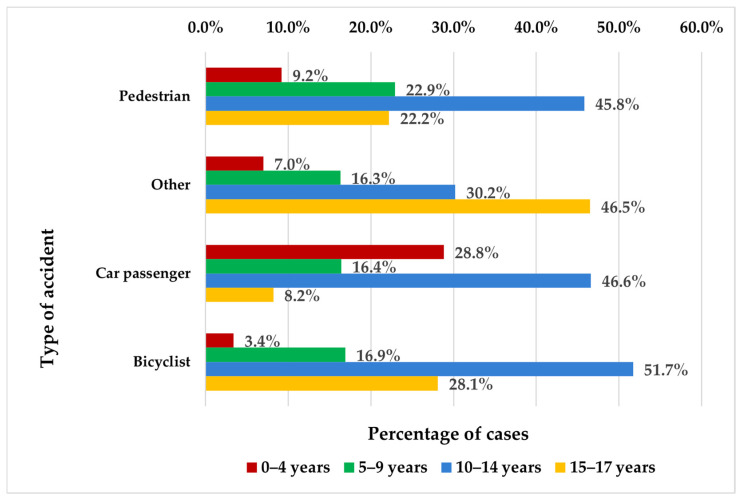
Patient distribution by age group and injury type.

Children aged 0 to 4 years were most commonly involved as car passengers, significantly more than as pedestrians, cyclists, or participants in other types of injuries (28.8% compared to 9.2%, 7%, and 3.4%, respectively). In contrast, adolescents aged 15 to 17 years were more often injured in other types of injuries or while cycling than as pedestrians or vehicle passengers (46.5% and 28.1% versus 22.2% and 8.2%, respectively) (*p* < 0.001) (Figure 1).

Age plays a significant role in determining the type of injury sustained (*p* < 0.001). For example, the probability of being injured as a car passenger decreases significantly with age. The regression coefficient for this category is −0.129 (*p* < 0.001), with an Exp (B) value of 0.879, indicating a 12.1% decrease in odds for each additional year of age compared to the reference group—pedestrians. In contrast, the likelihood of being involved in other types of injuries rises with age. This is reflected by a coefficient of 0.53, which is also statistically significant (*p* < 0.001). The corresponding Exp (B) of 1.703 suggests that with each additional year, the odds of experiencing this type of injury increase by approximately 70.3% relative to pedestrians. However, age does not significantly influence all injury categories.

There was a statistically significant association between age group and injury type (*p* < 0.001), indicating that certain injury types were more frequent in specific age categories. On the contrary, gender did not demonstrate a statistically significant association with the type of injury (0.09). This suggests that the distribution of injury types is comparable between males and females in this study (Table 1).

### 3.4. Associated Injuries—General Overview

Most of the patients included in our study had associated injuries, with a total of 798 cases (74.3%). The most frequently observed injuries were excoriations in 193 cases (24.1%), thoracic contusions in 130 cases (16.4%), abdominal contusions in 118 cases (14.8%), additional fractures in 86 cases (10.8%), traumatic brain injuries (TBI) in 82 cases (10.2%), limb contusions in 68 cases (8.5%), and hematologic alterations in 62 cases (7.8%). The remaining 7.4% consisted of a diverse range of injuries and conditions, such as spinal injuries, hemoperitoneum, traumatic shock, acute ischemia syndrome, hematoma, ecchymosis, gross hematuria, burns, sprains or dislocations accompanied by localized edema and hemarthrosis.

### 3.5. Specific Injury Patterns

#### 3.5.1. Traumatic Brain Injury (TBI)

There was no statistically significant association between abdominal contusions and accident type (*p* = 0.09), indicating that the mechanism of injury did not substantially influence their occurrence (Table 2). Similarly, no significant association was observed between abdominal contusions and sex (*p* = 0.59), suggesting that gender was not a determining factor. However, a significant association was found with age group (*p* = 0.03), with children aged 0–4 years experiencing a higher rate of abdominal contusions than those aged 10–14 years, as confirmed by Dunn–Bonferroni post hoc analysis (Table 3).

#### 3.5.2. Thoracic Contusions

**Figure 2:**
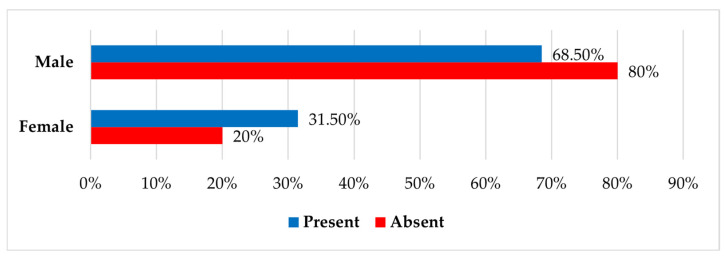
Distribution of patients by sex and presence of associated thoracic contusions.

Thoracic contusion rates did not differ significantly across injury categories (*p* = 0.11) (Table 3). Nevertheless, a statistically significant difference (*p* = 0.04) was observed when classified by sex, with females experiencing thoracic contusions at a higher rate than males (31.5% vs. 20%) (Figure 2 and Table 4).

There was no statistically significant association between the incidence of thoracic contusions and age group (*p* = 0.22) (Table 5).

#### 3.5.3. Abdominal Contusions

Fisher’s exact test revealed no statistically significant differences in the frequency of abdominal contusions across injury types (*p* = 0.59) or between sexes (*p* = 0.11), indicating that neither crash mechanism nor gender had a substantial impact on their occurrence. However, a significant age-related variation was identified, with children aged 0–4 years experiencing a higher rate of abdominal contusions compared to those aged 10–14 years. This difference was confirmed by Fisher’s exact test (*p* = 0.03) and further validated through Dunn-Bonferroni post hoc analysis (Table 6 and Table 7).

#### 3.5.4. Blood Count Abnormalities

**Figure 3:**
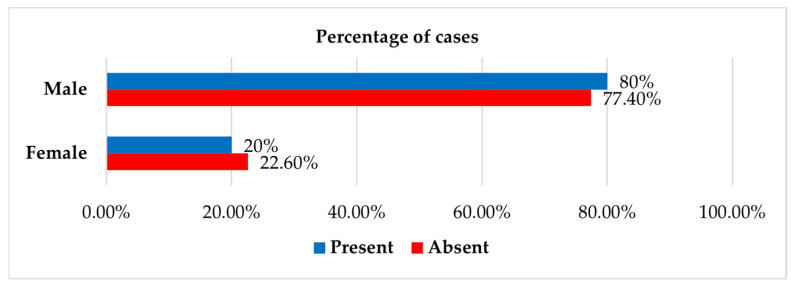
Distribution of patients by sex and presence of associated blood count changes.

Fisher’s exact test (*p* = 0.003) revealed a significant association between injury type and the occurrence of blood count changes, with Bonferroni-adjusted Z-tests showing that cyclists were more frequently affected (50% vs. 22.6%). In contrast, there was no statistically significant association with sex (*p* = 1.000) (Table 8 and Figure 3).

#### 3.5.5. Limb Contusions

**Figure 4:**
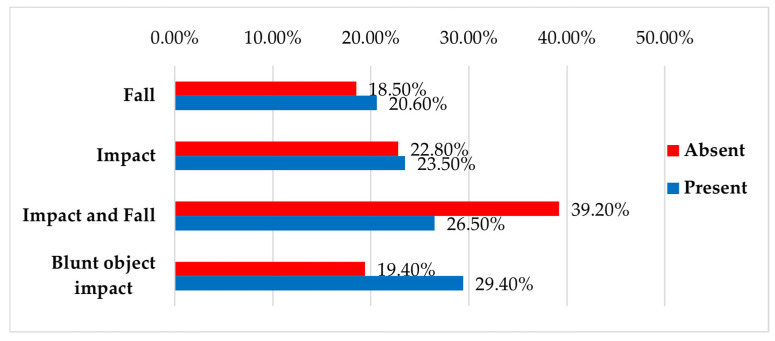
Distribution of patients by injury mechanism and presence of associated limb contusion injuries.

There was no statistically significant association between the occurrence of limb contusions and injury mechanism (*p* = 0.39), sex (*p* = 0.67), or age group (*p* = 0.19) (Figure 4 and Table 9).

#### 3.5.6. Excoriations

There was no statistically significant association between the occurrence of excoriations and patient sex (*p* = 0.68). In contrast, a significant difference was found across age groups (*p* < 0.001). Post hoc Dunn–Bonferroni analysis showed that excoriations were significantly less frequent in the youngest children (6.5% vs. 13.8%) and in older adolescents (12.5% vs. 28.3%), whereas children aged 10–14 years had a markedly higher prevalence (57.7% vs. 40.6%) (Table 10).

**Figure 5:**
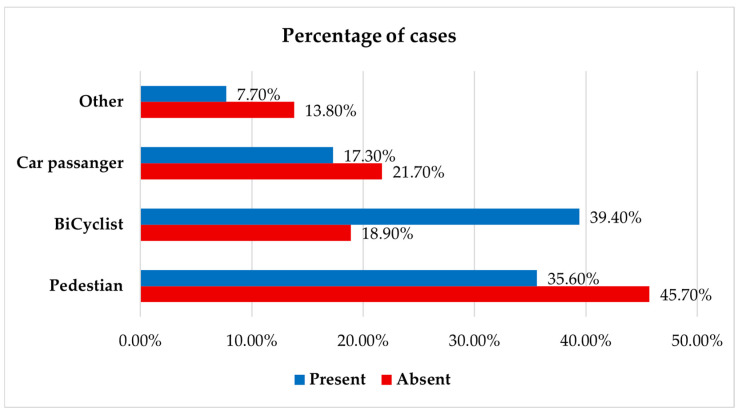
Distribution of patients by injury type and presence of associated excoriations.

Patients injured as cyclists exhibited excoriations at a markedly higher rate than other groups (39.4% versus 18.9%; *p* = 0.001) (Figure 5).

### 3.6. Protective Measures

Among the 163 pediatric patients identified as car passengers, restraint status was documented in 89.6% of cases: 57.7% were seated unrestrained in the rear seat, 20.9% were held in the caregiver’s arms, and only 11.0% were appropriately secured using an age-appropriate child safety seat; restraint status was unknown in the remaining 10.4% of patients.

**Figure 6:**
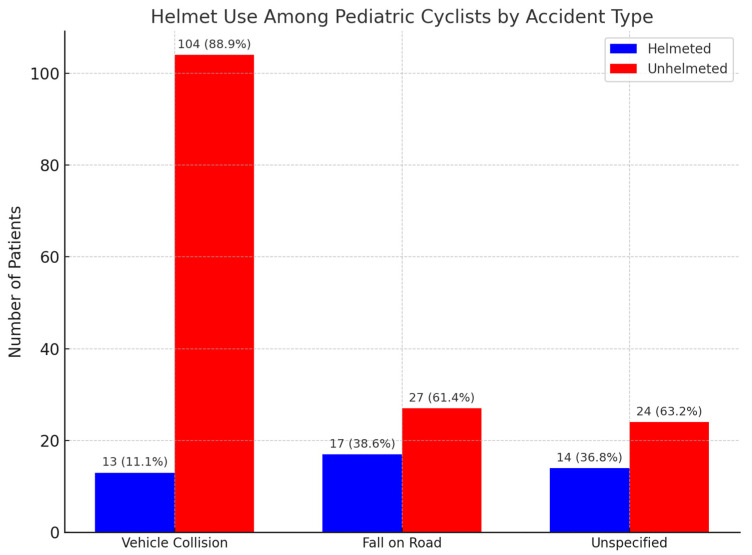
Helmet use among pediatric cyclists by accident type.

Among the 199 pediatric bicycle accident victims, 155 (78%) were not wearing helmets. Regarding accident type, 117 cases (58.8%) resulted from collisions with vehicles, 44 cases (22.1%) from falls on public roads, and 38 cases (19.1%) were unspecified. Helmet use was documented in only 13 vehicle collisions (11.1%), 17 falls (38.6%), and 14 unspecified incidents (36.8%). Both the absolute numbers and compliance rates indicate particularly low helmet use in high-energy crashes such as vehicle collisions. Within this cohort, 11 children (5.5%) sustained traumatic cranio-cerebral (TCC) injuries, with approximately 9 occurring among cyclists without a helmet and 2 among helmeted cyclists. No cases of coma were reported, suggesting that although head trauma was present, progression to severe neurological compromise was not observed (Figure 6).

## 4. Discussion

### 4.1. Global and Local Burden of Pediatric RTIs

The current study highlights that pediatric RTIs significantly impact child health globally, with most trauma cases involving males, likely due to greater exposure to high-risk environments and risk-taking behaviors [1,18]. Children aged 10–14 years had the highest injury rate, consistent with data from Poland and Turkey showing increased independence and traffic exposure in early adolescence [19,20]. These developmental trends support the need for age-focused preventive measures.

### 4.2. Age- and Sex-Specific Injury Patterns

A notable finding is the higher prevalence of thoracic contusions in female patients (31.5% vs. 20%). Possible explanations for the higher prevalence in females include anatomical differences in chest-wall compliance, seating position, and restraint use [21,22]. Further clarification might require biomechanical modeling or crash-simulation research [23,24].

Age-specific injury patterns also emerged: younger children (0–4 years) were more often injured as car passengers, while adolescents (15–17 years) experienced more injuries as cyclists or in other injury types [25,26]. This aligns with broader traffic injury trends and supports targeted intervention strategies [27,28].

### 4.3. Injury Mechanisms and Associated Trauma

Soft tissue injuries, such as excoriations and limb contusions, were the most frequent associated injuries in our study. While these are often classified as minor, they can serve as important markers for trauma severity. Pruksanubal et al. noted that such injuries may correlate with higher rates of traumatic brain injury (TBI) and should not be overlooked in pediatric trauma assessment [29]. This recommendation is further echoed by MacKay et al., who emphasized that patterns of limb injuries are often interconnected with age and injury mechanism [28].

Our findings revealed that the majority of excoriations occurred in children aged 10–14 years, which corresponds with findings by Kowalski et al. [19] and Iancu et al. [26], who observed increased vulnerability in this age group due to greater mobility and insufficient risk awareness. Moreover, cyclist-related injuries were more likely to be associated with hematological abnormalities, primarily decreases in hemoglobin and hematocrit indicative of blood loss, but in some cases also marked thrombocytopenia, aligning with the findings of Thompson et al. [30] and reflecting the high-energy nature of such injuries. While excoriations were the most frequent visible injuries in cyclists, these often occurred in the context of high-energy crashes, particularly collisions with motor vehicles. Such incidents produced not only superficial abrasions but also significant underlying trauma, which explains the frequent hematologic alterations observed in this group (e.g., anemia, thrombocytopenia). Excoriations should therefore be regarded as potential external markers of more severe systemic injury.

By contrast, abdominal contusions were more common in children aged 0–4, likely due to the anatomical vulnerability of internal abdominal organs and inappropriate restraint use [25,31]. Stylianos et al. further noted that young children’s developmentally sensitive abdominal region increases their risk of serious internal injury in collisions [31].

### 4.4. Demographic and Residential Context

Demographically, the mean age was 11.13 years, with boys representing 77.7% of cases and 64.8% of patients coming from Iași County, 76% of whom lived in rural areas. Although the national under-18 sex ratio is almost balanced (107 boys to 100 girls, or 51.7% vs. 48.3%) [32], the literature on injury involvement is mixed: many studies report higher male incidence, as seen in Weijermars et al. [33] and Elrud et al. [34], while Gustafsson et al. [35] observed risk variability based on age and sex. Our study found no statistically significant gender differences in injury type or frequency, suggesting other sociodemographic or behavioral factors may play a greater role.

Additionally, urban–rural differences were noted: males were more frequently injured in rural areas, while females predominated in urban contexts—possibly due to differences in supervision, behavior, and infrastructure access [36,37]. Ciobanu et al. highlighted that rural road safety in Romania remains a major challenge due to a lack of infrastructure and reduced enforcement of traffic laws [38].

### 4.5. Compliance with Safety Measures

Although Romanian legislation explicitly requires child restraint systems, compliance remains low. According to Article 97, paragraph 1, children under 135 cm in height may only travel in vehicles equipped with safety systems if secured in an approved child seat. In our cohort, most young passengers were either unrestrained or held by their caregivers (*n* = 50, 69.5%), rather than placed in appropriate safety seats [39]. Similarly, Article 160, paragraph 4, advises cyclists to wear certified helmets when traveling on public roads, yet 69.6% (62 of 89) in our sample were not wearing a helmet at the time of the crash.

In Romania, the minimum legal age for obtaining a driver’s license for cars (category B) is 18 years, which means that none of the pediatric cases classified as car passengers in our study were legally eligible to drive [40]. This is an important distinction, as it contrasts with regulations in some countries, such as the United States, where provisional driving permits are often granted from the age of 15 or 16.

### 4.6. Policy Implications and Safety Design

The higher rate of thoracic injuries in females has received limited attention in the literature. However, Kent et al. and Doud et al. suggest that physiological differences such as chest compliance and impact distribution may contribute [21,22]. Our findings support the need for sex-sensitive safety protocols, particularly in seatbelt design and placement. While producing entirely separate systems for boys and girls may be impractical and costly, targeted design refinements such as adjustable belt positioning, adaptable anchorage points, or additional padding could enhance safety without requiring completely different products.

To improve compliance, penalties should be designed to promote behavioral change without becoming overly punitive, supported by an effective enforcement framework. Legislative measures alone are insufficient—successful implementation often relies on advocacy groups, community engagement, and accessible safety equipment [41,42,43]. The consistent and mandatory enforcement of seatbelt and helmet use should be a public safety priority. Policymakers must carefully calibrate enforcement strategies, weighing the severity of fines against potential unintended consequences [44].

Among the 163 pediatric patients identified as car passengers, restraint status was documented in 89.6% of cases: 57.7% were seated unrestrained in the rear seat, 20.9% were held in the caregiver’s arms, and only 11.0% were appropriately secured using an age-appropriate child safety seat; restraint status was unknown in the remaining 10.4% of patients. These findings are concerning and highlight significant gaps in safety compliance, even in high-income settings such as Romania. Similar concerns have been echoed by Freeman et al., who stressed that seatbelt non-use dramatically increases both injury severity and legal consequences in pediatric trauma cases [45]. Furthermore, evidence suggests that placing children in the rear seat has led to a significant decline in airbag-related head and neck injuries, particularly since the 2000s—a trend that was also observed in our retrospective analysis.

Despite international campaigns and road safety frameworks such as the Vision Zero Road Safety Program and local policy initiatives [38,45,46], our findings suggest that enforcement and public education efforts still fall short, especially regarding child restraint use in vehicles. Expanding public health interventions to raise awareness and strengthen regulation enforcement could reduce preventable injuries among this vulnerable population.

### 4.7. Bicycle Safety and Helmet Use

In regard to bicycle safety and helmet use, we determined that 78% of the 199 pediatric cyclists were not wearing helmets at the time of injury. Among the 111 patients injured from vehicle collisions, only 11.1% wore helmets. For the 46 children who experienced injury after road falls, 30.4% had helmets, while 40.0% of those in the unspecified group (n = 42) were helmeted. This underlines a critical area of safety policy failure. Mitchell and Bambach have shown that helmet use significantly reduces pediatric cyclist injuries, including TBIs [47]. International studies have reported markedly higher rates of traumatic brain injury (TBI) among pediatric cyclists without a helmet, often exceeding 10–15% in severe crashes [44,47], highlighting the likely underestimation of neurological burden in our cohort and reinforcing the critical role of helmets in mitigating head trauma.

### 4.8. Methodological Considerations and Future Research

From a methodological standpoint, Imprialou and Quddus underscored the importance of data quality in crash research [31], a limitation acknowledged in our retrospective study. To overcome such constraints, Rueda-Domínguez et al. advocated for multicentric prospective designs [39], while Digby et al. and Matsui et al. proposed integrating machine learning and imaging in trauma prognosis [30,31]. Popa et al. also emphasized the medico-legal complexities of pediatric traffic trauma in Romania [30].

### 4.9. Broader Implications

The relevance of our findings extends beyond Romania. Albedewi et al. and Wesson et al. emphasized that pediatric traffic trauma is an under-addressed global concern, especially in rapidly developing regions [35,36]. Du et al. further illustrated how helmet legislation directly influences pediatric TBI outcomes, underlining the necessity of evidence-based public health policy [44].

These findings strongly support the implementation of age- and sex-specific prevention strategies: secure car restraint systems in toddlers, pedestrian safety education in elementary schools, helmet policies and safe cycling programs for pre-adolescents, infrastructure and behavioral safety campaigns for older adolescents [38,45].

## 5. Limitations of This Study and Further Studies

This study has several limitations inherent to its retrospective, single-center design. First, reliance on medical records led to incomplete or inconsistent documentation of key variables such as seatbelt and helmet use, injury dynamics, and injury mechanisms. Second, the absence of detailed crash scene data and pre-hospital information limited the ability to fully analyze contributing environmental or behavioral factors. Third, the findings may not be generalizable beyond the study region due to the localized population and healthcare infrastructure. Additionally, the lack of long-term follow-up precluded assessment of outcomes such as functional recovery or disability. Finally, unmeasured confounding variables—such as socioeconomic status, parental supervision, and road safety education—may have influenced both exposure to risk and injury severity.

## 6. Conclusions

This study highlights critical age- and gender-specific patterns in pediatric road traffic injuries, with a predominance of unrestrained passengers and underuse of protective equipment like helmets and car seats. Children aged 10–14 years and males were most commonly affected, though significant thoracic trauma was more frequent in females. The findings underscore the urgent need for targeted safety interventions, improved parental education, and stricter enforcement of child restraint regulations. Enhancing injury surveillance and integrating prospective, multicenter data could further inform effective prevention strategies.

## Figures and Tables

**Table 1:** Associations between age, gender, and injury type.

Type of Injury		Pedestrian		Bicyclist		Car Passenger		Other		*p* *
		*n*	%	*n*	%	*n*	%	*n*	%	
Gender	Female	98	28.8%	38	19.1%	29	17.8%	13	14%	0.09
	Male	242	71.2%	161	80.9%	134	82.2%	83	86%	
Age	0–4 years	31	9.2%	34	16.9%	47	28.8%	7	7%	
	5–9 years	78	22.9%	103	51.7%	27	16.4%	16	16.3%	<0.001
	10–14 years	156	45.8%	56	28.1%	76	46.6%	29	30.2%	
	15–17 years	74	22.2%	7	3.4%	13	8.2%	45	46.5%	

* Fisher’s exact test.

**Table 2:** Distribution of patients by type of injury and presence of associated TBI.

Injury Type	Absent		Present		*p* *
	*n*	%	*n*	%	
Pedestrian	153	41.5%	187	50%	0.09
Bicyclist	139	27.1%	60	11.5%	
Car passenger	75	19.9%	88	23.1%	
Other	41	11.4%	55	15.4%	

* Fisher’s Exact Test.

**Table 3:** Distribution of patients by gender, age, and presence of associated TBI.

TBI		Absent		Present		*p* *
		*n*	%	*n*	%	
Gender	Female	86	21.9%	98	25%	0.59
	Male	313	78.1%	301	75%	
Age	0–4 years	35	10.5%	56	17.3%	0.03
	5–9 years	72	19%	84	21.2%	
	10–14 years	203	47.1%	160	36.5%	
	15–17 years	91	23.5%	99	25%	

* Fisher’s exact test.

**Table 4:** Distribution of patients by injury type and presence of associated thoracic contusions.

Injury Type	Absent		Present		*p* *
	*n*	%	*n*	%	
Pedestrian	143	39.6%	197	54.8%	0.11
Bicyclist	119	26.7%	80	17.8%	
Car passenger	96	21.8%	67	15.1%	
Other	47	11.9%	49	12.3%	

* Fisher’s exact test.

**Table 5:** Distribution of patients by age and presence of associated thoracic contusions.

Age	Absent		Present		*p* *
	*n*	%	*n*	%	
0–4 years	40	10.9%	51	13.7%	0.22
5–9 years	80	19.6%	74	17.8%	
10–14 years	203	47.7%	160	37%	
15–17 years	78	21.8%	112	31.5%	

* Fisher’s exact test.

**Table 6:** Distribution of patients by injury type and presence of associated abdominal contusions.

Injury Type	Absent		Present		*p* *
	*n*	%	*n*	%	
Pedestrian	160	41.7%	180	47.1%	0.59
Bicyclist	117	26.4%	82	18.6%	
Car passenger	78	20.1%	85	21.4%	
Other	46	11.8%	50	12.9%	

* Fisher’s exact test.

**Table 7:** Distribution of patients by gender, age, and presence of associated abdominal contusion injuries.

Abdominal Contusion		Absent		Present		*p* *
		*n*	%	*n*	%	
Gender	Female	75	20.5%	109	30%	0.11
	Male	325	79.5%	289	70%	
Age	0–4 years	29	9.4%	62	20%	0.03
	5–9 years	83	19.8%	73	17.1%	
	10–14 years	211	48.3%	152	34.3%	
	15–17 years	84	22.6%	106	28.6%	

* Fisher’s exact test.

**Table 8:** Distribution of patients by type of injury and presence of associated blood count changes.

Type of Injury	Absent		Present		*p* *
	*n*	%	*n*	%	
Pedestrian	210	44.2%	130	26.7%	0.003
Bicyclist	62	22.6%	137	50%	
Car passenger	124	21.6%	39	6.7%	
Other	39	11.6%	57	16.7%	

* Fisher’s exact test.

**Table 9:** Distribution of patients by gender, age, and presence of associated limb contusion injuries.

Limb Contusion Injuries		Absent		Present		*p* *
		*n*	%	*n*	%	
Gender	Female	103	22.8%	81	17.6%	0.67
	Male	295	77.2%	319	82.4%	
Age	0–4 years	74	12.3%	17	2.9%	0.19
	5–9 years	88	19.8%	66	14.7%	
	10–14 years	149	43.8%	214	61.8%	
	15–17 years	103	24.1%	87	20.6%	

* Fisher’s exact test.

**Table 10:** Patient distribution by age, gender, and presence of excoriations.

Excoriations		Absent		Present		*p* *
		*n*	%	*n*	%	
Gender	Female	128	22.6%	53	23%	0.68
	Male	438	77.4%	179	77%	
Age	0–4 years	62	12.5%	29	5.8%	<0.001
	5–9 years	68	17.4%	86	23%	
	10–14 years	149	40.5%	214	58.7%	
	15–17 years	133	28.6%	57	12.5%	

* Fisher’s Exact Test.

## Data Availability

The data presented in this study are available on reasonable request.
